# Experimental Research on a Hybrid Algorithm for Localisation and Reconstruction of the Impact Force Applied to a Rectangular Steel Plate Structure

**DOI:** 10.3390/s22218123

**Published:** 2022-10-24

**Authors:** Binbin Qiu, Yang Lu, Xianqiang Qu, Xu Li

**Affiliations:** 1 School of Mechanical Engineering, University of Shanghai for Science and Technology, Shanghai 200093, China; 2 College of Shipbuilding Engineering, Harbin Engineering University, Harbin 150001, China; 3 Aerospace Science & Industry Defence Technology Research and Test Center, Beijing 100854, China

**Keywords:** impact force identification, hybrid algorithm, PRMCSM method, optimisation process, Tikhonov regularisation

## Abstract

Impact force is the most common form of load which acts on engineering structures and presents a great hidden risk to the healthy operation of machinery. Therefore, the identification or monitoring of impact forces is a significant issue in structural health monitoring. The conventional optimisation scheme based on inversion techniques requires a significant amount of time to identify random impact forces (impact force localisation and time history reconstruction) and is not suitable for engineering applications. Recently, a pattern recognition method combined with the similarity metric, PRMCSM, has been proposed, which exhibits rapidity in practical engineering applications. This study proposes a novel scheme for identifying unknown random impact forces which hybridises two existing methods and combines the advantages of both. The experimental results indicate that the localisation accuracy of the proposed algorithm (100%) is higher than that of PRMCSM (92%), and the calculation time of the hybrid algorithm (179 s) for 25 validation cases is approximately one nineteenth of the traditional optimisation strategy (3446 s).

## 1. Introduction

The knowledge of load (including static and dynamic) excitation applied to mechanical systems is crucial for many practical engineering problems such as strength analysis, vibration isolation, fault diagnosis, and structural health monitoring [1,2,3,4,5,6,7,8]. As the most common form of dynamic load, impact force is the main cause of material fatigue for many steel structures; for fibre-reinforced composite structures, it may cause spalls, pits, cracks, delamination, and even failure. The most famous failure is the Space Shuttle Columbia disaster, in which the composite tile on the leading edge of the wing fractured due to impact. Therefore, it is crucial to understand the characteristics of external loads such as impact location and time history. Generally, the external load can be measured directly by placing a force transducer on the force transfer path; however, due to the high economic cost and the change of structural characteristics, it is not desirable to obtain load information by direct measurement. In addition, impact forces often act in unpredictable or inaccessible locations that are difficult to directly measure. In recent years, research on indirect identification and measurement of impact forces has increased exponentially [1,2,3]. Due to the effects of impact forces on structural integrity and operational safety, their identification has been the subject of many studies. However, it remains a challenging inverse problem that is always associated with ill conditions, and small errors in structural responses may lead to large deviations between reconstructed and actual solutions. Therefore, inverse analytical techniques are proposed to reformulate ill-posed problems into well-posed problems [2,3].

The focus of this study is to acquire information on impact forces in time and space domains. Therefore, the current research status of impact force identification is presented in this paper. When the impact location is known, many recent scholars have proposed several new methods to reduce ill-posed impact force reconstruction procedures and obtain stable and reliable solutions. Zhang et al. [9] presented a Bayesian approach for force reconstruction which can deal with both measurement noise and model uncertainty. Ding et al. [10] proposed a discrete force identification method based on an average acceleration discrete algorithm in state space, and the external excitation that acts on a structure was estimated with a regularisation method. By introducing Bellman’s principle of optimality into the dynamic programming equation, a new method was proposed in [11] to identify the time history of the input excitation, which can eliminate large fluctuations in the identified results. Liu et al. [12,13] proposed two new methods, namely, the shape function method, which was based on moving least squares fitting, and the interpolation method, which effectively reduced the ill-posedness and steadily identified the dynamic force. Samagassi et al. [14] proposed a wavelet relevance vector machine approach to reconstruct multiple impact forces. Prawin and Rao [15] presented an online input force time history reconstruction algorithm and an optimal sensor placement algorithm. In considering the sparse nature of impact force in the time domain, Qiao et al. [16,17] developed two general group sparse regularisation methods by minimising mixed *l*_2,1_-norm and *l*_1_-norm for the inverse problem of impact force identification. Yan [18] employed the Bayesian inference regularisation to solve the ill-posed impact force reconstruction problem. Chang et al. [19] proposed an implicit Landweber method for single-source and multi-source force reconstructions, and the response sensitivity was utilised to reconstruct the time history of dynamic force. Qiu et al. [20] proposed a novel criterion, called the local convex curve, to realise accurate reconstruction of the impact force time history by suppressing the tail disturbance of the reconstructed time history.

In practical applications, establishing how to obtain the impact location rapidly and accurately is an important problem which needs to be addressed intensively. In the past decades, many investigations have been conducted in the field of impact force localisation. One of the most common approaches to determine the impact location is based on the triangulation technique, in which at least three sensors are mounted for signal collection and the velocity of the wave is essential. For a simple structure with isotropic materials, the triangulation method can be applied directly based on a constant wave speed in all directions. However, for composite materials or homogeneous complex structures, which are usually anisotropic, the wave propagation speeds in all directions are different and should be measured. Hajzargerbashi et al. [21] proposed a new objective function and a new source location algorithm to simplify the optimisation procedure based on triangulation techniques, and the performance was experimentally verified for a homogeneous anisotropic plate and a non-homogeneous anisotropic plate. Zhao et al. [22] proposed an integrated impact localisation scheme using the arrival time obtained by wavelet transform analysis, in which the triangulation localisation technique together with the proposed hybrid algorithm based on particle swarm optimisation and a genetic algorithm were employed. Yang [23] used the normalised signal energy instead of the wave arrival time in the triangulation method to estimate the impact location. An innovative method for real-time estimation of the impact position on concrete structures by utilising the P-wave signals collected by lead PZT sensors was proposed by Zhu et al. [24]. Jang et al. [25] proposed a novel scheme based on the triangulation method to localise impacts on a composite stiffened panel; neural network training was adopted by using normalised signal magnitudes as the inputs and the distance of impact from each sensor as the output. In summary, the triangulation technique is limited and does not exhibit good prospects for engineering applications.

As the most popular scheme, the optimisation process localises the impact force by minimising the residual error function between the simulated and measured structural responses [26]. Li and Lu [27] proposed a two-step approach to both localise and reconstruct the single point force acting on a structure: the first step is to optimise the error function through a complex method to localise the force, and the second step is to reconstruct the force history according to classic regularisation strategy. Wambacq et al. [28] presented an algorithm for the localisation of forces which involved minimising an objective function that was penalised with a group sparsity term and solved by the interior point method. Kalhori et al. [29] developed an inverse method to estimate the location and magnitude of impact forces under the assumption that a number of impact forces were simultaneously acting on all potential locations but that only one impact force produced an effect. The normalised residual norm and Chebyshev polynomial were proposed by Hu [30] to reconstruct the impact time history and estimate the force position. Some scholars [31,32] introduced the Maxwell–Betti Reciprocity Theorem to the problem of localisation of impact forces and achieved good results by improving the objective function in the optimisation process. Furthermore, Liang et al. [33] proposed a distributed coordination algorithm to quickly and effectively localise the impact, based on the inverse analysis method and triangulation method, which can solve the impact localisation problem in parallel.

Among these strategies, the first strategy has limited scope for practical applications, whereas the second strategy is time-consuming for the localisation of random impact forces. In addition to the two traditional impact force localisation strategies, other novel methods have been proposed to solve this problem. Artificial intelligence is the focus of information technology development, and is applied to estimate the impact force location [34,35,36,37]. Furthermore, Thiene and Galvanetto [38] presented an innovative algorithm to detect the position of impact on composite plates, in which the proper orthogonal decomposition of the dynamics of the system after impact was used without the information of wave propagation. Kalhori et al. [39] proposed two strategies based on a similarity searching technique to simultaneously identify both the location and magnitude of the impact force acting on a rectangular composite panel. Lage et al. [40] presented a method for identification of the locations of the forces applied to a structure by using the concept of response transmissibility. To realise the reconstruction and localisation of impact loads, Jayalakshmi [41] formulated the associated inverse analysis as a constraint optimisation problem and then proposed a hybrid adaptive differential search algorithm, which has proven to be robust and effective. Qiu et al. [42] proposed a method to quickly acquire the positions of random impacts by using the cosine similarity of the structural stress responses, which significantly reduced the computational effort and improved the localisation accuracy. Furthermore, the PRMCSM method was verified by a model experiment and a new similarity metric index was presented with the same properties as the cosine similarity [43].

In this study, an innovative hybrid algorithm is proposed to localise random unknown impact forces. To localise impact forces rapidly, the feasibility of the PRMCSM method has been demonstrated by numerical simulation [42] and model experiment [43]. However, according to the experimental data, the localisation of the impact forces is not completely satisfactory. Meanwhile, for the considered localisation-and-reconstruction problem, the optimisation process is time consuming, though it offers high precision. Grafting the two algorithms to improve the positioning accuracy and efficiency of random impact forces would be an attractive solution. The hybrid algorithm is created based on this idea, in which the basic strategy of the PRMCSM method is utilised to achieve region localisation of the random impact forces, and then the temporal and spatial information on the random impact loads can be achieved by using the small-scale optimisation process. Furthermore, some influencing factors of the proposed algorithm are discussed in this paper.

The innovative characteristics of this approach are summarised below:

- The proposed algorithm maintains high localisation accuracy and efficiency simultaneously in comparison to the traditional optimisation strategy and PRMCSM method.
- This algorithm is not demanding in terms of region delineation and is highly adaptable.

The remainder of this paper is organised as follows. In Section 2, Section 3 and Section 4, we present the conventional optimisation algorithm based on regularisation techniques, the basic theory of PRMCSM, and the theoretical background of the novel hybrid algorithm, respectively. Section 5 introduces the experimental setup. The experimental validation of the proposed approach will be highlighted and discussed in Section 6. Finally, Section 7 lists the conclusions of this study.

## 2. Optimisation Algorithm for Impact Force Identification

As a typical second type inverse problem, the aim of impact force identification is to obtain unknown force vectors based on mathematical models and measured data. We usually consider the dynamic excitation within a linear elastic system as a superposition of impulses in the time domain and express the system response as the convolution of the excitation with the transfer function [1,3]. If the initial conditions for a linear time-invariant system satisfy y(0)=0 and $\dot{y}(0)=0$, the structural response can be expressed as

(1) $$y(t)=g(t)⊗p(t)=\int_{0}^{t}g(t-\tau )p(\tau )d\tau$$

where y(t) can be dynamic responses of the structure, g(t) represents the Green kernel function or impulse response function, and p(t) denotes the dynamic force.

However, in practice it is necessary to discretise the continuous convolution in the time domain, and the discretisation form is detailed in Equation (2). In this equation, Δt denotes the time increment, while n denotes the number of time-domain partitions. The symbol $t_{j}$ indicates the *j*^th^ time point, which is equal to jΔt (j=1,2,⋯,n).

(2) $$y(t_{j})=\Delta t\sum_{i}^{j}g(t_{j}-\tau_{i})p(\tau_{i})$$

Furthermore, Equation (2) can be transformed into the matrix-vector form, as shown in Equation (3); further simplification leads to its compact form, as shown in Equation (4).

(3) $$\left[\begin{array}{c} y(t_{1})\\ y(t_{2})\\ \vdots \\ y(t_{n}) \end{array}\right]=\left[\begin{array}{cccc} g(t_{1}) & 0 & \cdots & 0\\ g(t_{2}) & g(t_{1}) & \ddots & \vdots \\ \vdots & \vdots & \ddots & 0\\ g(t_{n}) & g(t_{n-1}) & \cdots & g(t_{1}) \end{array}\right]\left[\begin{array}{c} p(t_{1})\\ p(t_{2})\\ \vdots \\ p(t_{n}) \end{array}\right]\Delta t$$

(4) Y=G⋅P

Equations (3) and (4) are the fundamental governing equations for force identification. In order to obtain a stable and reliable solution, Tikhonov regularisation and L-curve criterion [26] are chosen to solve this problem.

In the optimisation process, the residual error function between the simulated and measured responses of all the sensors is minimised for all potential impact force locations, and the location and time history can then be obtained simultaneously [26]. Therefore, the objective function is expressed as Equation (5).

(5) $$d_{j}=\sum_{i=1}^{n}\left\|G_{ij}\cdot P_{j}-Y_{i}\right\|$$

Here, $Y_{i}$ is the response vector of the *i*th measurement point, $P_{j}$ is the simulated impact force vector at the *j*th loading point, $G_{ij}$ is the Green kernel function matrix between the *j*^th^ loading point and the *i*th measurement point, and n is the number of sensors.

The normalised residual error norm, instead of the residual error norm, is applied to determine the position of the impact force [30]. It represents the proportion of the response deviations to the measured responses. In this paper, the residual error function is utilised for the optimisation process, as shown in Equation (6).

(6) $$d_{j}=\frac{\sum_{i=1}^{n}\left\|G_{ij}\cdot P_{j}-Y_{i}\right\|}{\sum_{i=1}^{n}\left\|Y_{i}\right\|}$$

## 3. PRMCSM Algorithm for Impact Force Identification

As an important statistical pattern recognition tool, discriminant analysis is associated with many disciplines and can be used in many fields. Its core idea is to draw the laws from samples of known classes, then establish discriminant formulae and standard, and determine the classes of the new samples. The most commonly used discriminant analysis methods include K-nearest neighbour discriminant, distance discriminant, Bayesian discriminant, and so on. The PRMCSM method is a typical distance-based discrimination method. As an important fundamental concept in multivariate statistical analysis, distance has different mathematical meanings according to different definitions.

According to [42], impact localisation and reconstruction are the two most important parts of the identification procedure on impact force identification. The localisation of impact force involves four steps, which are summarised as data acquisition, feature extraction, region localisation, and force localisation. As the focus of their study, feature extraction was highlighted prominently. A similarity metric can be used to reveal the similarity degree of several discrete time series and the similarity searching technique based on this index can be utilised to find the most similar time series. As an intrinsic characteristic of the structure, the transfer functions between the impact forces and sensors can be employed to identify the impact force. Based on the linear assumption that response depends linearly on external excitation, if the positions of the forces are closer, the transfer functions are more similar. Thus, based on this property, the similarity metric can be used to determine the impact position. As a unique similarity metric, cosine similarity uses the cosine of the angle between two non-zero vectors to represent the error between them without involving the vector magnitudes. To some extent, cosine similarity translates the similarities of the transfer functions into similarities of the structural responses [42].

Based on the feature calculation method mentioned in [42], the formulae for calculating the feature values for region and force localisations can be harmonised as Equation (7).

(7) $$C_{i,j-k}=d(y_{ij},y_{ik},cosine),\;(i=1,2,\cdots ,n;\;k=1,2,\cdots ,m)$$

where n denotes the number of acceleration sensors and j represents all the reference and validation points; k denotes the central reference point in each divided area and m represents the number of selected central reference points. Furthermore, the term cosine in the equation represents cosine similarity.

Afterwards, we can calculate the feature vectors used for pattern recognition by using Equation (8) and obtain the training and validation databases according to [42].

(8) $$FeatureVector_{j}=[C_{1,j-1},\cdots ,C_{n,j-1},C_{1,j-2},\cdots ,C_{n,j-2},\cdots ,C_{1,j-m},\cdots ,C_{n,j-m}]$$

As mentioned above, all of the impact forces exerted to the same small area are grouped into one class and the region division inevitably results in many classes in this study. For a multi-class discrimination problem, the determination of the discriminant functions is crucial. If we assume that M classes exist in the n-dimensional space, then their corresponding discriminant functions are $d_{1}$, $d_{2}$, …, and $d_{M}$. If $X=(x_{1},\;x_{2},\;\ldots ,\;x_{n}{)}^{T}$ belongs to Class *i*, then Equation (9) would be yielded.

(9) $$d_{i}(X)>d_{j}(X)\;\mathrm{or}\;d_{i}(X)<d_{j}(X),\;(j=1,2,\cdots ,M;i\neq j)$$

Therefore, region localisation can be accomplished by using this multi-class discrimination strategy, in which a similarity classifier based on the Euclidean distance discriminant function as well as these constructed feature vectors is utilised and rapidly determines the approximate action region of the impact forces and eliminates the majority of the disturbing factors.

To lock the exact impact location, the combination vector $D_{j}$, shown in Equation (10), is proposed based on the acceleration responses $y_{ij}$.

(10) $$D_{j}=[y_{1j}^{T},y_{2j}^{T},\cdots ,y_{nj}^{T}]$$

The combination vectors for the reference and validation points in the same small area are obtained using Equation (10), and we can identify the nearest reference points based on their cosine similarities. Therefore, the problem of localising the nearest reference point can be converted into a cosine-similarity-based pattern matching problem in which each class contains only one sample.

After completing the random impact force localisation, a general procedure based on the fundamental control equation for impact force time history, introduced in Section 2, is employed, in which different regularisation techniques and regularisation parameter criteria can be used. Moreover, the transfer matrices of the target positions (nearest reference points) are established according to the experimental data and impact time histories are reconstructed by them instead of the actual transfer matrices.

## 4. Hybrid Algorithm Based on Optimisation Process and PRMCSM Method

A novel hybrid algorithm based on the optimisation process and PRMCSM method is proposed in this study, and this method can not only meet the requirement of location accuracy but also take into account the efficiency of location identification. It is well known that, according to the conventional optimisation scheme described in Section 2, the identification of the impact force for a finite degree of a freedom system or continuous structure is time consuming, but offers the guarantee of high accuracy. Meanwhile, the PRMCSM method displays good performance in terms of rapidity and robustness. Maintaining the accuracy and efficiency of impact force localisation is the most significant highlight of the proposed hybrid algorithm, in which hybridisation of the two existing methods is adopted. The conceptual framework of the proposed method originates from the simple idea that, under the conditions of many pre-set impact force positions, fewer potential locations are considered if the possible range of unknown impact forces is locked over a small area in advance, thus the time of determining impact force position and time history through the optimisation process will be greatly reduced. The PRMCSM scheme was proposed to pay attention to the identification of impact forces with random positions and the main step of this scheme is region localisation, which determines the approximate action region of the impact forces. Therefore, the hybrid algorithm uses region localisation technology based on pattern recognition to determine the scope of impacts, and then uses the optimisation process based on normalised residual error norm and the Tikhonov regularisation strategy to simultaneously realise accurate impact force localisation and time history reconstruction.

Based on the conceptual framework of the hybrid algorithm, the four steps of the whole process for random impact force identification can be summarised as follows:

(1) Step one: Pre-processing

A survey region with uniformly distributed reference points and randomly generated verification points is set up, where the impact force is applied. The corresponding experimental data (including acceleration and impact force signals) are recorded and used in the following steps.

(2) Step two: Region localisation

Set the smallest divided region size, divide the survey region continuously, and use the region localisation strategy (including feature extraction and pattern recognition) to find the target region where the random impact load is located.

(3) Step three: Transfer matrix construction

The transfer matrices of all reference points on the locked region are established based on the Tikhonov method.

(4) Step four: Random impact force identification

The localisation and time history reconstruction of random impact forces are realised based on the optimisation scheme and Tikhonov method.

**Figure 1:**
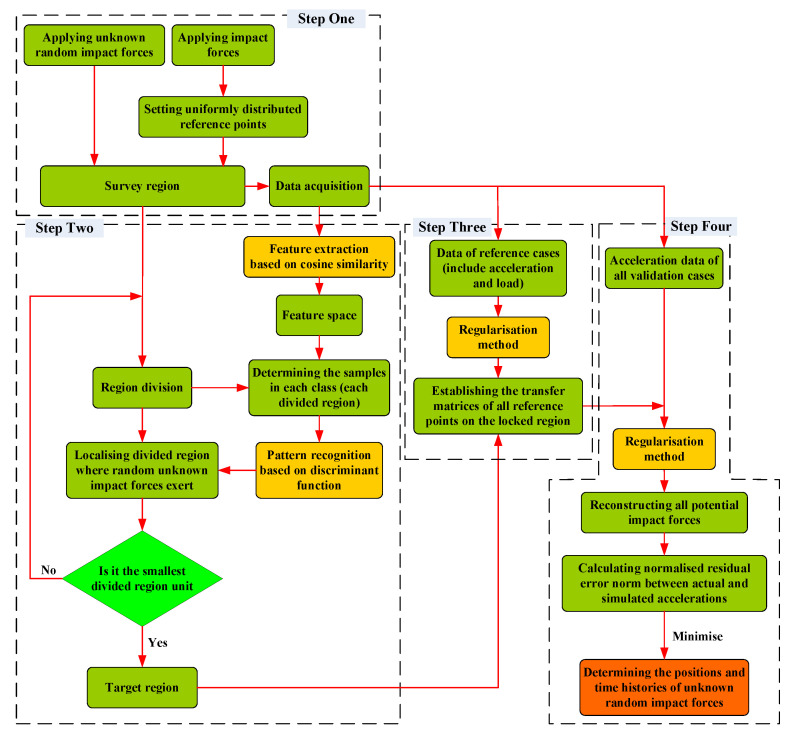
The process for random impact force identification based on a hybrid algorithm.

The detailed flow of the hybrid method is illustrated in Figure 1 and some of the specification details will be introduced in the next section along with the experimental model adopted in this study.

## 5. Experimental Set-Up and Procedure

Due to the financial and experimental restrictions, a rectangular steel plate available in the laboratory was chosen to validate the proposed hybrid method. The details of the experimental model are listed in Table 1, and the distributions of sensors and impact points are also mentioned in this part.

**Figure 2:**
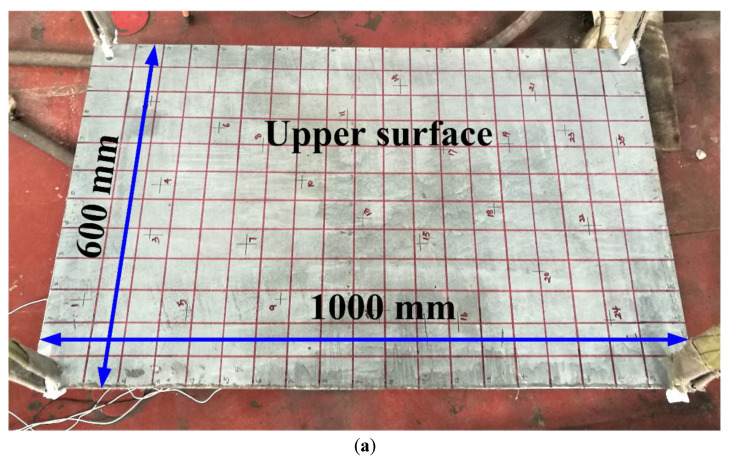

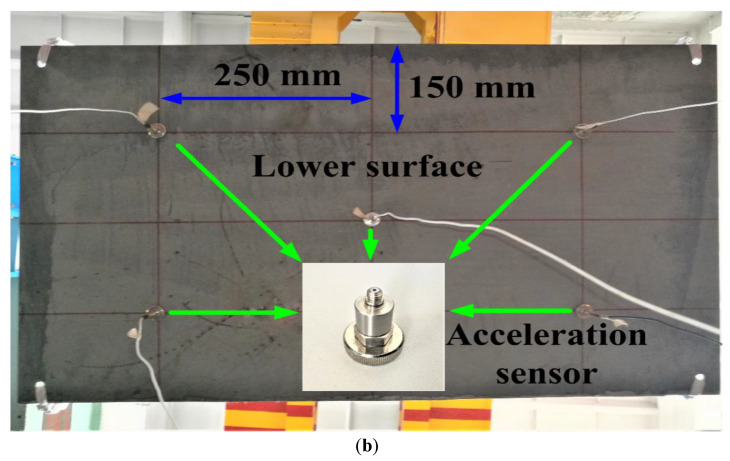
Upper surface (**a**) and lower surface (**b**) of rectangular steel plate.

As illustrated in Figure 2, four suspend round holes were set at the four vertices of the rectangular steel plate and four soft ropes were used to suspend this experimental piece in mid-air. All impact forces were applied to its upper surface by employing a hammer with an aluminium tip. Meanwhile, due to experimental convenience and the high signal-to-noise ratio, five unidirectional acceleration sensors were chosen to be attached to the lower surface to collect the acceleration responses in the vertical direction. The manufacturers and types of force hammer and acceleration sensors were described in [43]. In Figure 2b, all edges of the lower surface are divided into four equal parts, and according to the sensor placement criterion proposed in [43], the acceleration sensors are mounted at the cross points, which are not simultaneous on the symmetry axis of the rectangular steel plate.

**Figure 3:**
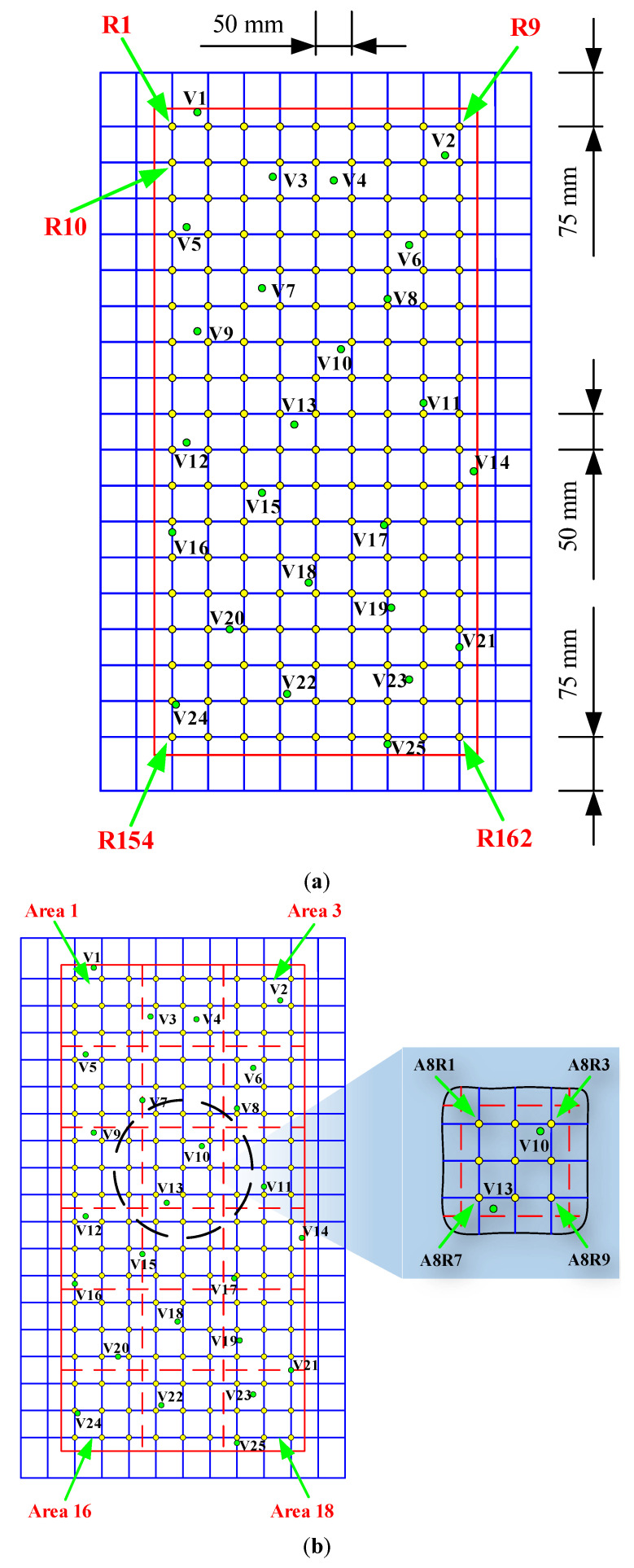

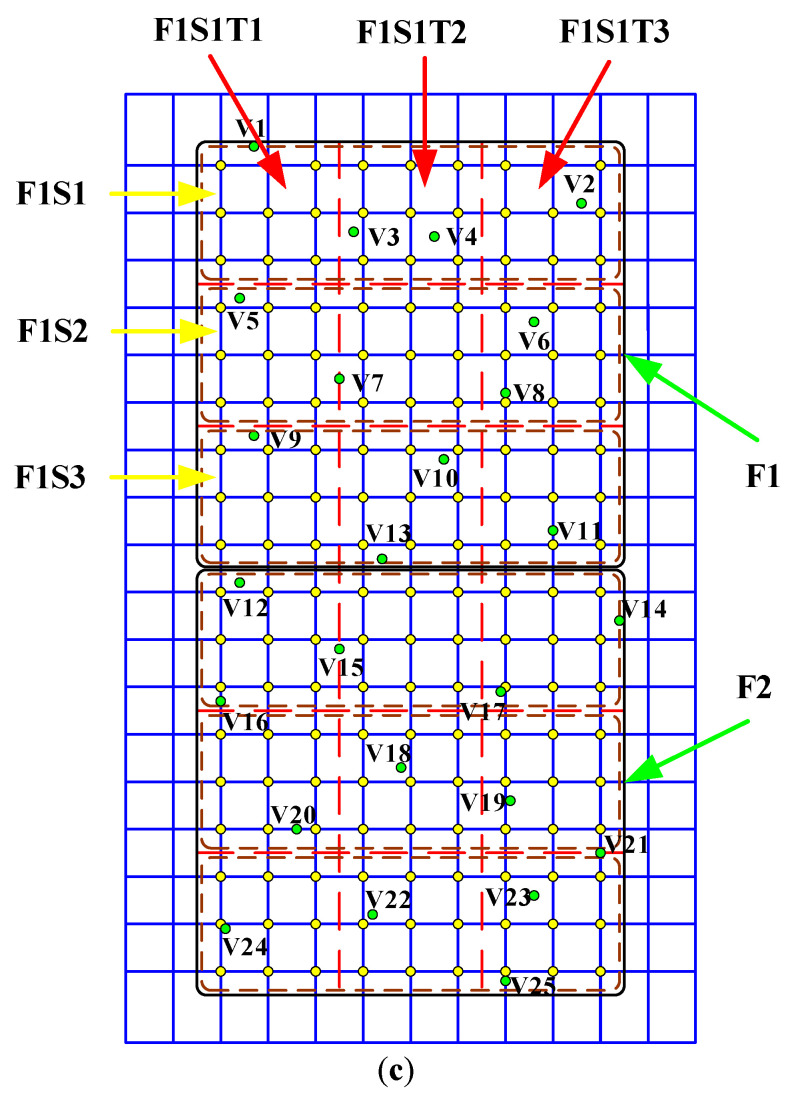
Setting of region divisions, reference points, and validation points for the (**a**) optimisation algorithm, (**b**) PRMCSM algorithm, and (**c**) hybrid algorithm.

Due to the four hanging holes, the survey region enclosed by the red solid lined box is at a certain distance from the upper and lower boundaries of the plate. A certain number of seeds were disposed on the surrounding boundary of the rectangular steel plate and then a grid was constructed according to the distribution of seeds, which was described in our previous study. Details are available in Figure 3a. Therefore, there were 162 grid nodes (reference points) in the selected survey region where impact forces were applied. For different approaches, the naming rules of the reference points are different. For the conventional optimisation algorithm, the reference points are marked in a left-to-right and top-to-bottom order, as shown in Figure 3a. In addition, 25 randomly generated green points on the survey region represented the validation points. The serial numbers of all validation points are also specified in Figure 3a.

However, for the PRMCSM algorithm and the proposed hybrid algorithm, the survey region should be segmented due to the introduction of the classification concept. According to the division rule proposed in [42,43] for the PRMCSM algorithm, there are 18 small areas in the survey region presented in Figure 3b and nine reference points that are evenly distributed on each small area. The first small area in the upper left corner of the survey region is denoted as “Area 1”, and the divided areas are also marked in a left-to-right and top-to-bottom order. Similar to the naming rule of the areas, the nine reference points in each small area are designated as R1–R9 and are arranged in a left-to-right and top-to-bottom order. In order to distinguish the reference points of different areas, they are renamed by adding the letter A and the area number, which is shown in the partially enlarged image of Figure 3b.

In this study, three survey region division strategies are proposed for the hybrid algorithm according to the technological process mentioned in Section 4, and the smallest divided region unit is equivalent to the small area described in the region division for the PRMCSM scheme. For the first strategy, the survey region is divided into two parts: F1 and F2. Then, each part is divided into three sub-parts, and the suffix “S” and the serial number of the sub-parts are added on the basis of the first division, such as F1S1. From Figure 3c, it is evident that each sub-part is composed of the three smallest region units, and referring to the naming rules for the second region division, each smallest region unit is named in the format F1S1T1. Furthermore, the letters “F”, “S”, and “T” represent the first, second, and third divisions, respectively. For the second strategy, if the first division in the first strategy is not performed, the subsequent two region divisions are directly carried out, which means that the survey region is divided into six strip-shaped sub-regions, each of which is composed of the three smallest region units. Similarly, the third division strategy directly divides the survey region into the smallest region units, which is the same as the region division of the PRMCSM algorithm. Due to the restriction in the article length, the schematic diagrams of the latter two region divisions are not shown here.

According to the characteristics of the pattern recognition in PRMCSM and hybrid algorithms, the impact forces acting on the same area are considered to be one class. Determining the range of impacts quickly is the essential common aspect of these two methods and the only difference between them is how they can each accurately localise the impact forces in order to determine the nearest reference points.

**Figure 4:**
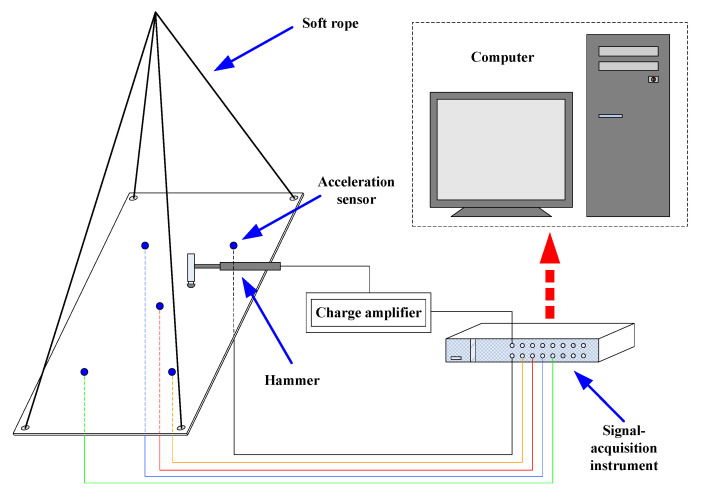
Schematic diagram of the impact experiment.

In order to introduce the relevant experimental instruments and connection settings, it is necessary to exhibit the impact experiment’s signal acquisition system (shown in Figure 4) in detail. The acceleration signals obtained from accelerometers were collected by a data acquisition instrument (NI PXI-1045), and the hammer sensor was linked to the same device by a charge amplifier (Denmark B&K Company (Nærum, Denmark), 2692). Since the head of the hammer was hemispherical, it was assumed that the impact forces were perpendicular to the rectangular steel plate and that the impact would not lead to material degradation. LabView Signal Express software was utilised to complete the relevant settings of the acceleration sensors and data acquisition, and all the acceleration and load data collected by the data acquisition instrument were aggregated into the computer and eventually transformed into text files by using NI DIAdem software. The sampling frequency was 100 kHz. Furthermore, all the algorithms employed herein were validated with MATLAB 2011a; the CPU of the computer used was Intel I7-8550U, with 8 GB RAM.

## 6. Analysis and Discussion

This section concerns the feasibility, effectiveness, and accuracy of the hybrid algorithm and verification of the proposed scheme from different perspectives through a model experiment. In order to illustrate the superiority of the proposed method, this section is divided into three parts. In the first part, the results of localisation and time history reconstruction using the hybrid method are analysed. Afterwards, in the next part, the application of different region divisions to the hybrid method to reveal the influence of this key factor on identification accuracy is discussed and an analysis based on a comparison of the performances is presented. Finally, the performance of the hybrid method is compared with those of the conventional and PRMCSM methods to reveal its significant advantages.

### 6.1. Experimental Verification of the Hybrid Algorithm

**Figure 5:**
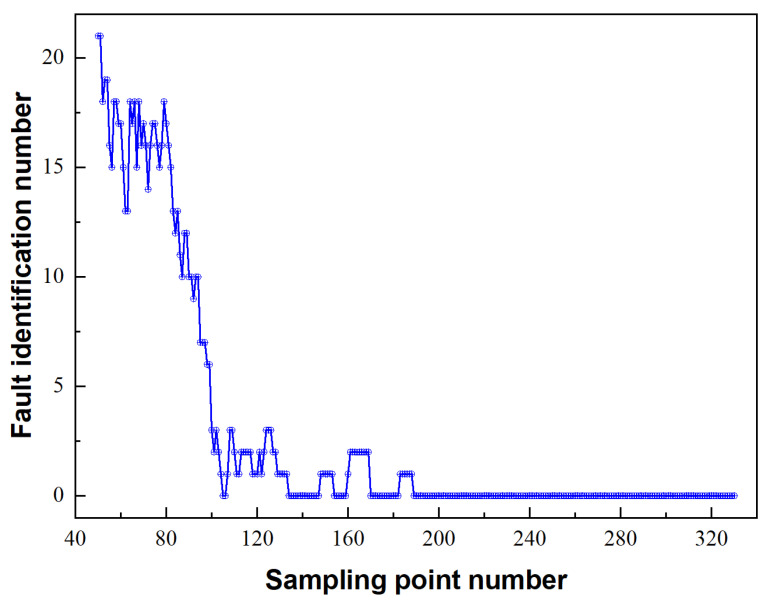
FIN versus SPNs for region localisation through cosine similarity.

As mentioned above, for the hybrid algorithm, it is clear that there are two parts in the proposed scheme, region localisation and force localisation, that are based on the pattern recognition method and optimisation process, respectively. For region localisation, feature extraction is an indispensable step. Therefore, before analysing the results of impact force identification by using the hybrid algorithm, it is important to discuss the selection of the sampling point number (SPN). Since the hypothesis of impact forces (all dynamic calculations are performed under the linear assumption and all impact forces have the same shape and different amplitudes) introduced in [42] suffers from inherent defects, the conclusions regarding the SPN still need to be verified by experiments. Moreover, SPN is only used in the region localisation part of the hybrid algorithm due to the existence of the optimisation process in the hybrid scheme. According to the feature extraction for region localisation, the results for the fault identification number (FIN) under different SPNs are presented in Figure 5. Using the SPN for region localisation as the horizontal coordinate and the FIN as the vertical coordinate, the localisation results by the hybrid algorithm based on three region divisions are obtained. It is apparent that, with the increase in the SPN, the FIN decreases notably, which has a monotonous decreasing appearance. When the number of sampling points reaches 190, the region identification results tend to be stable. However, such results conflict with the numerical simulation results observed in [42]. In their research, the SPN for region localisation is evidently optimal. This may be attributed to the small number of experimental samples in this paper, which suggests that there is a strong contingency; however, the average FIN is too small to be ignored and a wide selection range can be more suitable for practical engineering applications. In the subsequent algorithm validation process, 200 sampling points are utilised for region localisation, which is larger than 190.

After completing the selection of sampling points, it is essential to discuss the identified results of the impact forces by the hybrid method in detail, including the time histories and locations of the random impact forces. Depending on the previous relevant experimental settings, there are 25 validation cases in this study and the determined impact locations are listed in detail in Table 2. The symbol “√” indicates correct localisation. It is evident that the hybrid method identifies all 25 random impact forces correctly, and its localisation accuracy reaches 100%. However, it is essential to discuss the localisation results of all of the validation cases in detail, and the 25 validation cases can be divided into two categories: specific cases and common cases. In addition, several special cases are further subdivided into two subcategories. The first one is that when the location of the impact occurs at the centre of the mesh, it indicates that there are four reference points closest to the impact position, any of which can be considered as a target candidate (validation points V4 and V7). The second subcategory is the location where the impact acts along the mid-perpendicular line of the mesh edge, which indicates that the two nearest reference points can be deemed as correct targets (validation points V15 and V21). The impact force localisation results of the four special cases at the critical positions are completely correct, which, together with the results of the normal case, indicates the reliability of the proposed method in terms of location identification.

Although the results of impact force localisation obtained using the proposed algorithm are prominently satisfactory, it is essential to evaluate the reconstructed impact force time history. Therefore, the peak relative error (PRE), relative error (RE), and correlation coefficient (CC) between the actual force $P_{\mathrm{actual}}$ and the reconstructed force $P_{\mathrm{reconstructed}}$ are defined as Equations (11)–(13):

(11) $$PRE=\frac{\left|\max(P_{\mathrm{reconstructed}})-\max(P_{\mathrm{actual}})\right|}{\max(P_{\mathrm{actual}})}$$

(12) $$RE=\left\|\frac{P_{\mathrm{reconstructed}}-P_{\mathrm{actual}}}{P_{\mathrm{actual}}}\right\|$$

(13) $$CC=\frac{\sum_{i=1}^{N}\left[p_{\mathrm{reconstructed}}(t_{i})-E(P_{\mathrm{reconstructed}})\right]\left[p_{\mathrm{actual}}(t_{i})-E(P_{\mathrm{actual}})\right]}{{\left[P_{\mathrm{reconstructed}}-E(P_{\mathrm{reconstructed}})\right]}^{T}\left[P_{\mathrm{actual}}-E(P_{\mathrm{actual}})\right]}$$

where $\left|\cdot \right|$ represents the absolute value of a scalar and max(⋅) represents the maximum of a vector.

**Figure 6:**
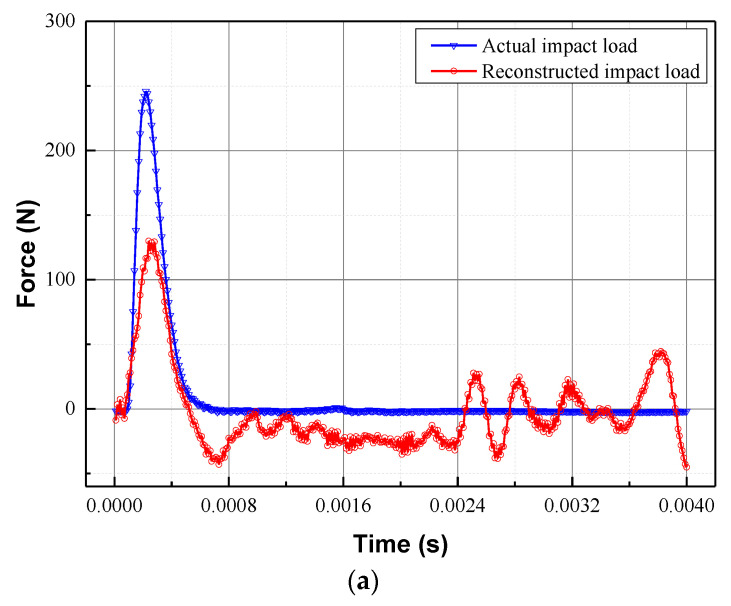

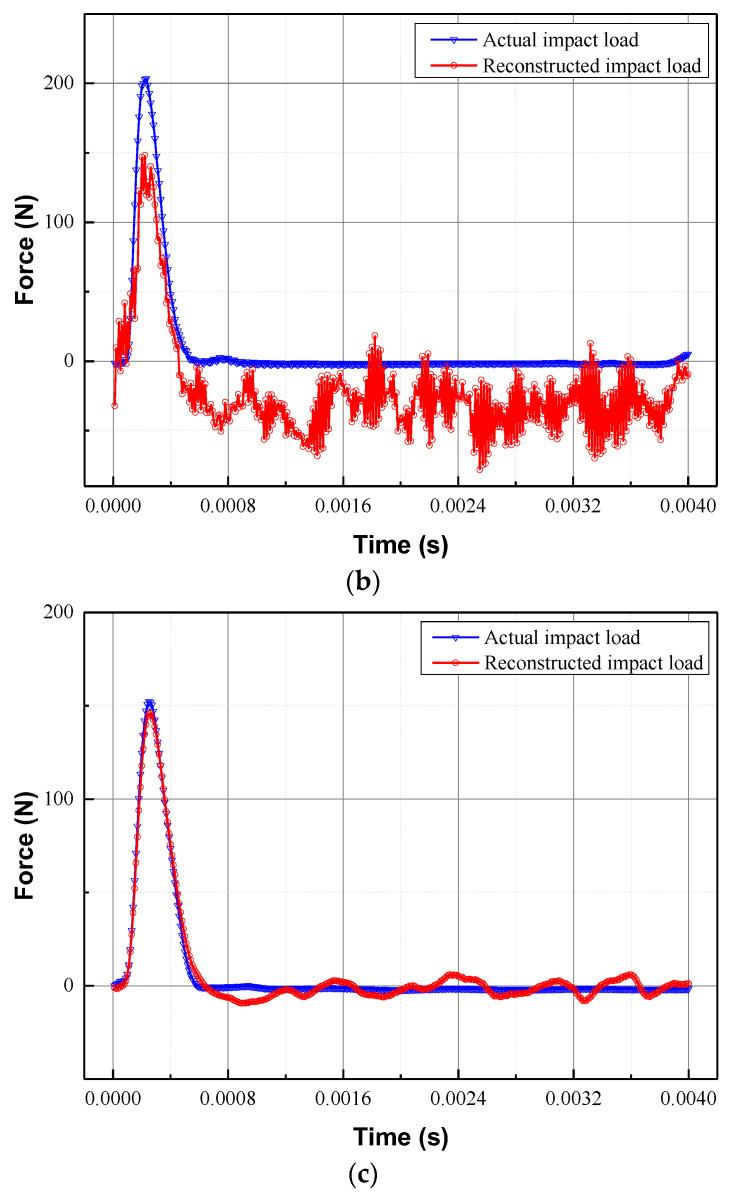
Reconstruction of the impact forces at (**a**) V4, (**b**) V20, and (**c**) V24 validation points.

Table 2 provides not only the results for the identified locations, but also the PREs, REs, and CCs of all of the validation cases. It is evident that the hybrid algorithm can completely localise the random impact forces of the 25 validation cases. Therefore, the location information does not need to be discussed much, and the focus should be shifted to the reconstruction of time history. Noticeably, the maximum PRE (47.13%) and the smallest CC (0.8233) are observed in validation case V4, while the maximum RE (93.28%) is noticed in validation case V20. In order to illustrate the reconstruction results, the reconstructed impact forces for the validation cases V4, V20, and V24 are depicted in Figure 6a,c, respectively.

It is evident that the objective existence of peak difference between the actual and simulated impact forces means that the validation case with the maximum peak relative error shown in Figure 6a is barely satisfactory. So how do we improve the reconstruction accuracy of the impact forces? Our first thought is to reduce the distance between the target reference point and the validation point. If the validation point coincides with the target reference point, the identification of the random impact force degrades to the identification of the position-determined impact force. However, due to the random nature of the impact location, reducing the mesh size becomes an effective means to improve accuracy, which means a suitable grid density can help overcome the disadvantage of the proposed method. Furthermore, the high correlation coefficients suggest that the basic strategy of this study is entirely feasible. For validation case 20, the reconstructed impact force is significantly lower than the actual impact force and is accompanied by significant fluctuations due to noise amplification and error accumulation in the inversion process. However, in most cases, the hybrid algorithm performs well, as shown in Figure 6c for V24.

**Table 2:** Identification results obtained through the hybrid algorithm.

Validation Point	Nearest Reference Point	Localisation	PRE	RE	CC
V1	F1S1T1R2	F1S1T1R2 (√)	0.3165	0.4281	0.9307
V2	F1S1T3R6	F1S1T3R6 (√)	0.1370	0.2840	0.9569
V3	F1S1T2R4	F1S1T2R4 (√)	0.1017	0.3403	0.9451
V4	F1S1T2R5, F1S1T2R6, F1S1T2R8, or F1S1T2R9	F1S1T2R9 (√)	0.4713	0.6342	0.8233
V5	F1S2T1R1	F1S2T1R1 (√)	0.2523	0.4537	0.9385
V6	F1S2T3R2	F1S2T3R2 (√)	0.3519	0.7315	0.9590
V7	F1S2T1R6, F1S2T1R9, F1S2T2R4, or F1S2T2R7	F1S2T2R7 (√)	0.3611	0.4641	0.9150
V8	F1S2T3R7	F1S2T3R7 (√)	0.0167	0.1704	0.9887
V9	F1S3T1R2	F1S3T1R2 (√)	0.1563	0.2240	0.9779
V10	F1S3T2R3	F1S3T2R3 (√)	0.2500	0.4057	0.9354
V11	F1S3T3R8	F1S3T3R8 (√)	0.1551	0.3095	0.9506
V12	F2S1T1R1	F2S1T1R1 (√)	0.1375	0.3421	0.9491
V13	F1S3T2R7	F1S3T2R7 (√)	0.3551	0.8485	0.8822
V14	F2S1T3R6	F2S1T3R6 (√)	0.2125	0.3760	0.9333
V15	F2S1T1R6, or F2S1T2R4	F2S1T1R6 (√)	0.3531	0.4341	0.9453
V16	F2S1T1R7	F2S1T1R7 (√)	0.0807	0.2087	0.9799
V17	F2S1T3R7	F2S1T3R7 (√)	0.0590	0.1496	0.9927
V18	F2S2T2R5	F2S2T2R5 (√)	0.0824	0.2379	0.9727
V19	F2S2T3R4	F2S2T3R4 (√)	0.1831	0.2676	0.9690
V20	F2S2T1R9	F2S2T1R9 (√)	0.2710	0.9328	0.8591
V21	F2S2T3R9, or F2S3T3R3	F2S2T3R9 (√)	0.0155	0.3012	0.9542
V22	F2S3T2R4	F2S3T2R4 (√)	0.0262	0.1692	0.9907
V23	F2S3T3R2	F2S3T3R2 (√)	0.3209	0.3984	0.9449
V24	F2S3T1R4	F2S3T1R4 (√)	0.0409	0.1321	0.9907
V25	F2S3T3R7	F2S3T3R7 (√)	0.0490	0.1824	0.9821

### 6.2. Accuracy and Efficiency Analysis Based on Different Region Divisions

After completing the verification of the hybrid algorithm, it is necessary to discuss the influence of different region divisions for the hybrid algorithm and whether their performances (including accuracy and efficiency) reveal obvious differences. Three survey region division strategies have been introduced in detail in the previous section on the experimental set-up, therefore, three hybrid algorithms based on direct one-region division (minimum region units), two-region divisions, and three-region divisions are obtained by using the concept of the proposed hybrid algorithm, which are represented as “hybrid algorithm (1)”, “hybrid algorithm (2)”, and “hybrid algorithm (3)”, respectively. These hybrid algorithms were utilised to determine the positions of the random impacts and the obtained results of all of the validation cases presented in this subsection. It is apparent that the three hybrid algorithms based on different region divisions determine the positions of all unknown random impact forces (the nearest reference points), which means that the accuracies of the three proposed algorithms reach 100%. Furthermore, to eliminate the influence of contingency, a MATLAB program was run 20 times for all the validation cases and the total calculation time for each run is recorded, though this has not been included in this paper. However, the average total calculation times of the 25 validation cases for “hybrid algorithm (1)”, “hybrid algorithm (2)”, and “hybrid algorithm (3)” are presented in Table 3, which are 179.53 s, 179.19 s, and 179.38 s, respectively. This suggests that obtaining the location information and time history of each random impact force will take about 7.2 s, which indicates that there are highly obvious engineering application prospects. Therefore, a conclusion can be easily drawn in that, for region localisation of impact force by using the pattern recognition method, as long as the smallest region unit is the final target, multiple region divisions will not affect the localisation accuracy and the error between the efficiencies can be ignored.

### 6.3. Comparative Analysis of Different Methodologies

After completing the feasibility study of the hybrid algorithm and discussing the possible influence factors, it is essential to analyse the advantages and disadvantages of the hybrid algorithm in detail and compare it with the traditional optimisation algorithm and PRMCSM algorithm. The locked impact force locations of the 25 validation cases obtained by using the optimisation algorithm, PRMCSM algorithm, and hybrid algorithm are listed in Table 4. Detailed information on nearest reference points for different algorithms can be found in the Appendix A. It is evident that the optimisation algorithm and hybrid algorithm completely lock the nearest reference point of each validation case; meanwhile, the PRMCSM algorithm mis-localises two random impact forces. This indicates that the localisation accuracy of the PRMCSM method (92%) is less than those of the optimisation and hybrid algorithms (100%). It is necessary to compare the identification results of these three algorithms. In particular, it should be noted that there are still two cases (V13 and V20) where the impact force cannot be localised correctly using PRMCSM. In such failed identification cases, the two inaccurate spots are the second closest reference points. Due to the discussion above and the high localisation accuracy, the results of the optimisation and hybrid algorithms are not discussed much.

In addition to the localisation results of random impact forces, the calculation times for obtaining the location and time history of the impact forces based on the different algorithms are also an important consideration factor. If the calculation efficiency is high, the algorithm is more likely to be used to localise and reconstruct the random impact forces rapidly in engineering practice. It takes 3446 s to complete the acquisition of temporal and spatial information for 25 unknown random impacts by using the optimisation algorithm, whereas it only takes 22 s and 179 s with the PRMCSM and hybrid algorithms, respectively. This means that each validation case requires 137.84 s, 0.88 s, and 7.16 s with the optimisation, PRMCSM, and hybrid algorithms, respectively. For hybrid algorithms, such short times for the identification of the impact forces meets the practical requirements of engineering applications. Therefore, generally speaking, the hybrid algorithm is an algorithm with a higher accuracy than the PRMCSM algorithm and a higher efficiency than the optimisation algorithm, and it exhibits greater application prospects.

## 7. Conclusions

This study proposes a novel scheme for completing the task of unknown random impact force identification, which is a hybridisation of the conventional optimisation algorithm and the PRMCSM algorithm and combines the advantages of these two algorithms. Furthermore, the proposed hybrid method was verified through a steel plate, and its performance indicates that this method displays prospects for extremely important engineering applications. Some conclusions were drawn as follows:

- A feasibility study of the hybrid algorithm was conducted based on a hanging rectangular plate model and the experimental results indicate that the proposed method exhibits a high accuracy in the determination of the locations of random impact forces and an acceptable requirement in the reconstruction of time histories.
- For region localisation of impact forces based on the hybrid algorithm, once the smallest region unit is determined, multiple region divisions will not affect the localisation accuracy or efficiency. However, this was only verified by using a simple plate structure, without involving a complex structure.
- Compared with the traditional optimisation algorithm, the hybrid algorithm offers the same accuracy, but its computational time is much less than that of the former; meanwhile, compared with that of the PRMCSM algorithm, its efficiency is slightly lower, though its accuracy is higher. Therefore, the hybrid algorithm maintains the accuracy and effectiveness of impact localisation, which is the most important highlight.

Though this work is devoted to the validation of the hybrid method, there are some influencing factors that need to be considered and verified in subsequent studies. The most typical one is the lack of quantitative analysis of grid density and it is necessary to discuss its effect on the accuracy of the proposed algorithm. Furthermore, the research object in this study was a two-dimensional steel plate which did not involve anisotropy and non-linearity. Therefore, it is not at all representative of engineering applications and the verification of more complex engineering structures should be taken into account.

## Figures and Tables

**Table 1:** Details of the experimental model.

Geometrical parameters	Length (mm)	1000
	Width (mm)	600
	Thickness (mm)	5
Material properties (Q235)	Young’s modulus (Pa)	2.06 × 10^11^
	Poisson’s ratio	0.3
	Density (kg/m^3^)	7850

**Table 3:** Results of hybrid algorithms based on different region divisions.

Algorithm Name	Hybrid Algorithm (1)	Hybrid Algorithm (2)	Hybrid Algorithm (3)
Average total calculation time (s)	179.53	179.19	179.38
Accuracy rate (%)	100%	100%	100%

**Table 4:** Identification results of validation cases obtained with different algorithms.

Validation Point	Localisation		
	Optimisation Algorithm	PRMCSM Algorithm	Hybrid Algorithm (3)
V1	R2 (√)	A1R2 (√)	F1S1T1R2 (√)
V2	R18 (√)	A3R6 (√)	F1S1T3R6 (√)
V3	R13 (√)	A2R4 (√)	F1S1T2R4 (√)
V4	R24 (√)	A2R8 (√)	F1S1T2R9 (√)
V5	R28 (√)	A4R1 (√)	F1S2T1R1 (√)
V6	R35 (√)	A6R2 (√)	F1S2T3R2 (√)
V7	R49 (√)	A5R7 (√)	F1S2T2R7 (√)
V8	R52 (√)	A6R7 (√)	F1S2T3R7 (√)
V9	R56 (√)	A7R2 (√)	F1S3T1R2 (√)
V10	R60 (√)	A8R3 (√)	F1S3T2R3 (√)
V11	R80 (√)	A9R8 (√)	F1S3T3R8 (√)
V12	R82 (√)	A10R1 (√)	F2S1T1R1 (√)
V13	R76 (√)	A8R8(×)	F1S3T2R7 (√)
V14	R99 (√)	A12R6 (√)	F2S1T3R6 (√)
V15	R93 (√)	A10R6 (√)	F2S1T1R6 (√)
V16	R100 (√)	A10R7 (√)	F2S1T1R7 (√)
V17	R106 (√)	A12R7 (√)	F2S1T3R7 (√)
V18	R122 (√)	A14R5 (√)	F2S2T2R5 (√)
V19	R124 (√)	A15R4 (√)	F2S2T3R4 (√)
V20	R129 (√)	A13R8(×)	F2S2T1R9 (√)
V21	R135 (√)	A15R9 (√)	F2S2T3R9 (√)
V22	R148 (√)	A17R4 (√)	F2S3T2R4 (√)
V23	R143 (√)	A18R2 (√)	F2S3T3R2 (√)
V24	R145 (√)	A16R4 (√)	F2S3T1R4 (√)
V25	R160 (√)	A18R7 (√)	F2S3T3R7 (√)
Correct number	25	23	25
Accuracy rate (%)	100%	92%	100%
Total calculation time (s)	3446	22	179

## Data Availability

Not applicable.

## Appendix A

**Table A1:** Nearest reference points of all validation cases obtained by different algorithms.

Validation Point	Nearest Reference Point		
	Optimisation Algorithm	PRMCSM Algorithm	Hybrid Algorithm (3)
V1	R2	A1R2	F1S1T1R2
V2	R18	A3R6	F1S1T3R6
V3	R13	A2R4	F1S1T2R4
V4	R14, R15, R23, or R24	A2R5, A2R6, A2R8, or A2R9	F1S1T2R5, F1S1T2R6, F1S1T2R8, or F1S1T2R9
V5	R28	A4R1	F1S2T1R1
V6	R35	A6R2	F1S2T3R2
V7	R39, R48, R40, or R49	A4R6, A4R9, A5R4, or A5R7	F1S2T1R6, F1S2T1R9, F1S2T2R4, or F1S2T2R7
V8	R52	A6R7	F1S2T3R7
V9	R56	A7R2	F1S3T1R2
V10	R60	A8R3	F1S3T2R3
V11	R80	A9R8	F1S3T3R8
V12	R82	A10R1	F2S1T1R1
V13	R76	A8R7	F1S3T2R7
V14	R99	A12R6	F2S1T3R6
V15	R93 or R94	A10R6 or A11R4	F2S1T1R6, or F2S1T2R4
V16	R100	A10R7	F2S1T1R7
V17	R106	A12R7	F2S1T3R7
V18	R122	A14R5	F2S2T2R5
V19	R124	A15R4	F2S2T3R4
V20	R129	A13R9	F2S2T1R9
V21	R135 or R144	A15R9 or A18R3	F2S2T3R9, or F2S3T3R3
V22	R148	A17R4	F2S3T2R4
V23	R143	A18R2	F2S3T3R2
V24	R145	A16R4	F2S3T1R4
V25	R160	A18R7	F2S3T3R7
